# Transhiatal esophagectomy in a high volume institution

**DOI:** 10.1186/1477-7819-6-88

**Published:** 2008-08-20

**Authors:** Andrew R Davies, Matthew J Forshaw, Aadil A Khan, Alia S Noorani, Vanash M Patel, Dirk C Strauss, Robert C Mason

**Affiliations:** 1 Department of general surgery, St Thomas' hospital, Guy's and St Thomas', NHS foundation trust, Lambeth Palace Road, London, SE1 7EH, UK

## Abstract

**Background:**

The optimal operative approach for carcinoma at the lower esophagus and esophagogastric junction remains controversial. The aim of this study was to assess a single unit experience of transhiatal esophagectomy in an era when the use of systemic oncological therapies has increased dramatically.

**Study Design:**

Between January 2000 and November 2006, 215 consecutive patients (182 males, 33 females, median age = 65 years) underwent transhiatal esophagectomy; invasive malignancy was detected preoperatively in 188 patients. 90 patients (42%) received neoadjuvant chemotherapy. Prospective data was obtained for these patients and cross-referenced with cancer registry survival data.

**Results:**

There were 2 in-hospital deaths (0.9%). Major complications included: respiratory complications in 65 patients (30%), cardiovascular complications in 31 patients (14%) and clinically apparent anastomotic leak in 12 patients (6%). Median length of hospital stay was 14 days. The radicality of resection was inversely related to T stage: an R0 resection was achieved in 98–100% of T0/1 tumors and only 14% of T4 tumors. With a median follow up of 26 months, one and five year survival rates were estimated at 81% and 48% respectively.

**Conclusion:**

Transhiatal esophagectomy is an effective operative approach for tumors of the infracarinal esophagus and the esophagogastric junction. It is associated with low mortality and morbidity and a five survival rate of nearly 50% when combined with neoadjuvant chemotherapy.

## Introduction

During the last thirty years, there has been a marked increase in the incidence of adenocarcinoma close to the esophagogastric junction whilst the incidence of squamous cell carcinoma of the esophagus has remained relatively unchanged [1]. Surgical resection of tumors in the esophagus and esophagogastric junction has been based upon the concept that, if all neoplastic tissue can be removed, a worthwhile period of survival and possibly cure can be achieved. Despite oncological advances, surgical resection is the only treatment that has repeatedly been shown to prolong survival, albeit in only 30% of patients [2].

Transhiatal esophagectomy is often advocated as the preferred surgical approach in patients with benign disease or early tumors or those patients with more advanced disease who would not tolerate a thoracotomy. This approach has been criticized because of the lack of a formal two field lymphadenectomy and the failure to completely resect the tumor under direct vision [2]. Transhiatal esophagectomy has been the favoured operative approach in our institution for managing both carcinoma of the oesophagus below the level of the carina and type I and II tumours of the esophagogastric junction. It has also been utilised for benign lower oesophageal disease including high grade dysplasia. This study evaluates our experience and outcomes with transhiatal esophagectomy in an era in which the use of neoadjuvant chemotherapy became more prevalent.

## Methods

### Study population

Between January 2000 and January 2007, 215 patients with benign or malignant disease of the intrathoracic esophagus and type I and II tumours of the esophagogastric junction underwent transhiatal esophagectomy at our institution. Prospective data on these 215 consecutive patients was collected from consultant databases supplemented by cancer registry data and case note review. A further 152 patients underwent transthoracic esophagectomy during the same time period and were excluded from analysis. Ethical committee approval was obtained for this study and the need for individual patient consent was waived.

### Preoperative evaluation and treatment

Routine preoperative evaluation involved upper gastrointestinal endoscopy with biopsy, endoscopic ultrasound and computed tomography of the neck, chest and abdomen. Staging laparoscopy and PET scanning were performed on a selective basis. Operative risk analysis included standard blood examination, electrocardiography, echocardiography, pulmonary function tests and cardiopulmonary exercise tests (in higher risk patients). Surgery was offered to medically fit patients following discussion at a multidisciplinary meeting.

90 patients in the study group (42%) received preoperative chemotherapy based upon the presence of T3 disease or positive lymph nodes on preoperative staging. The preferred chemotherapy at our institution consisted of three cycles of combination epirubicin, cisplatin and 5-fluorouracil each given over three weeks, following the MAGIC trial protocol [3].

### Operative technique

All patients underwent subtotal esophagectomy and proximal gastrectomy by the transhiatal technique as described in detail by Orringer. [4-6] An initial laparotomy was performed through a rooftop incision to confirm tumour resectability. After abdominal exploration and gastric mobilisation had been performed, the esophageal hiatus was enlarged by splitting the diaphragm anteriorly and retractors were positioned to facilitate exposure of the intrathoracic esophagus up to the level of the carina. This enabled en bloc resection of the esophagus and paraesophageal tissue including the crura and pleura (if indicated) under direct visualisation. Standard lymph node dissection involved lymph nodes in the lower mediastinum, around the esophagogastric junction and along the lesser curvature of the stomach. A radical lymph node dissection was performed at the origins of the left gastric and common hepatic arteries; lymph nodes at the celiac axis were included when enlarged and resectable. A less radical resection was performed for patients with benign disease. Gastrointestinal continuity was re-established with a narrow gastric tube vascularized by the right gastroepiploic artery in all cases, positioned within the posterior mediastinum. An end to side hand sewn single layer esophagogastric anastomosis was fashioned in the neck through a left sided cervical incision. Transmediastinal chest drains and placement of a feeding jejunostomy were performed in all patients.

### Pathological examination

Pathology specimens were processed by three dedicated esophagogastric pathologists according to Royal College of Pathologists' guidelines. [7] Tumors of the esophagogastric junction were categorized according to Siewert's classification based upon macroscopic tumor location, irrespective of the presence of Barrett mucosa. [8] Type I adenocarcinoma of the esophagogastric junction was staged according to esophageal pTNM classification whilst type II adenocarcinoma of the esophagogastric junction were staged according to gastric pTNM classification. [9] To ensure standardized histopathology results, all early specimens were re-categorized according to the latest guidelines.

### Follow up

During the immediate postoperative period, patients were kept intubated and ventilated until the following morning. Following extubation, patients were monitored on a surgical High Dependency Unit until well enough to be managed on a surgical ward. Oral nutrition was recommenced if a water soluble contrast swallow examination failed to demonstrate an anastomotic leak on the seventh day.

After discharge, patients were routinely followed up at 3–6 monthly intervals. Patients were offered either adjuvant chemotherapy (up to a maximum of 6 cycles) or chemoradiotherapy (if any margins were positive) based upon analysis of the pathological specimen and the histologically determined response to any preoperative treatment. Additional diagnostic procedures were only performed if indicated by the development of any new symptoms suggestive of recurrent disease. In the presence of recurrent disease, further oncological or palliative options were considered. The median duration of postoperative follow up was 26 months (range = 1–82 months) for all patients and 36 months (range = 2–82 months) for those alive at final follow up.

### Statistics

Overall survival was defined as the time interval from the date of operation until the date of death or most recent follow up. Disease free survival was defined as the time interval from the date of operation until the date of disease recurrence or most recent follow up. Survival curves were calculated according to the Kaplan-Meier method. Univariate group comparisons were calculated using the log rank test. Categorical variables were assessed using Fisher's exact test and continuous variables were assessed by student's t test [10]. A p value < 0.05 was regarded as statistically significant. Statistical analysis was performed with Graphpad Prism v3.0 and Instat v2.0 (GraphPad Software, San Diego California USA).

## Results

### Preoperative features

The demographic details of the 215 patients undergoing transhiatal esophagectomy are shown in Table 1. Dysphagia and weight loss were present in 73% and 48% of patients respectively with preoperatively confirmed malignant tumours. Twenty two patients (10%) had an asymptomatic cancer or high grade dysplasia detected during endoscopic surveillance of Barrretts oesophagus. Three patients (1%) underwent urgent transhiatal esophagectomy following endoscopic tumor perforation. According to the American Society of Anesthesiologists (ASA) classification [11], operative risk was scored as ASA-I (n = 15), ASA-II (n = 125), ASA-III (n = 72) or ASA-IV (n = 3).

**Table 1:** Demographic data on 215 patients undergoing transhiatal esophagectomy.

**Demographics**	**n**
Sex (M:F)	182:33
Age (range)	65 years (29–83 years)
	
**Preoperative indication**	
Adenocarcinoma	162 (75%)
Squamous cell carcinoma	23 (11%)
Other malignant tumours	3 (1%)
Benign tumours	1 (0.5%)
High grade dysplasia	23 (11%)
Benign strictures	3 (1%)
	
**Preoperative staging **(in 188 patients with preoperatively confirmed malignant tumours)	
T1	28 (15%)
T2	48 (26%)
T3	108 (57%)
T4	4 (2%)
N0	113 (60%)
N+	75 (40%)

#### Intraoperative surgical findings

Only one patient required intraoperative conversion to a right posterolateral thoracotomy due to tumor adherence at the carina and difficulties in achieving macroscopic tumor clearance through the esophageal hiatus. Macroscopic tumor clearance could not be achieved in one patient due to the presence of extensive left gastric and celiac axis lymphadenopathy. The median operative time was 151 minutes (range = 93–276 minutes).

#### Postoperative course

There were two in-hospital deaths during this study (<1%). One patient, a 74 year old man, with a past medical history including pneumonectomy for lung cancer and a previous myocardial infarction, developed respiratory failure requiring prolonged ITU admission and respiratory support; he died from myocardial infarction on day 44. The second patient, a 70 year old man, died from a pulmonary embolus on day 13 in ITU following admission with multiorgan failure secondary to chest sepsis.

Major postoperative complications are listed in Table 2. All 12 patients with clinically apparent anastomotic leaks were managed conservatively with opening the cervical wound to allow adequate wound drainage and reduction of oral intake combimed with jejunostomy tube feeding. None of these patients required re-operation for their anastomotic leaks. 10 patients (5%) required re-operation in the early post-operative stage for: bleeding (n = 4), bowel obstruction (n = 3), chyle leak (n = 2) and wound dehiscence (n = 1). Unplanned ITU admission was required in 29 patients (14%), most commonly for respiratory failure. The median ITU stay in this group was 7 days (range 2–44 days). Overall median length of hospital stay was 14 days (range 8–95 days). All patients were discharged directly home and the in-patient stay reflects the need for sufficient mobility and tolerance of an adequate oral diet prior to discharge.

**Table 2:** Major postoperative complications

**Complication**	**n (%)**
Clinical anastomotic leak	12 (5.6)
Respiratory^a^	65 (30)
Cardiovascular	31 (14)
Recurrent laryngeal nerve neuropraxia	6 (3)
Wound infection	22 (10)
Renal failure	6 (3)
Chyle leak	5 (2)
Deep vein thrombosis/pulmonary embolism	3 (1)

^a^Respiratory complications are defined as respiratory failure, lower respiratory tract infection and symptomatic pleural effusion requiring drainage.

### Oncological outcomes

Histopathological analysis of the operative specimens in the 215 patients revealed the following tumor types: adenocarcinoma (n = 169), squamous cell carcinoma (n = 22), high grade dysplasia (n = 17), adenosquamous carcinoma (n = 3), benign strictures only (n = 3) and spindle cell tumor (n = 1). In 3 patients, all initially diagnosed with adenocarcinoma, there was a complete pathological response to neoadjuvant chemotherapy whilst, in a further 2 patients, there was residual adenocarcinoma in lymph nodes only. The type of esophagogastric junctional tumour in 169 patients with adenocarcinoma was classified as follows: type I (n = 93), type II (n = 70) or type III (n = 6). All 6 patients with type 3 tumors had been preoperatively staged as type 2 tumours.

Macroscopic tumour clearance was achieved in 193 out of 194 patients with pathological evidence of invasive malignancy. Residual microscopic disease was found at the proximal or distal resection margins in 11 patients (5%), all in association with positive circumferential resection margins and involved lymph nodes. Eighty eight patients (46%) were subsequently found to have tumor cells at or within 1 mm of the esophageal adventitia or the gastric serosal surface.

**Figure 1:**
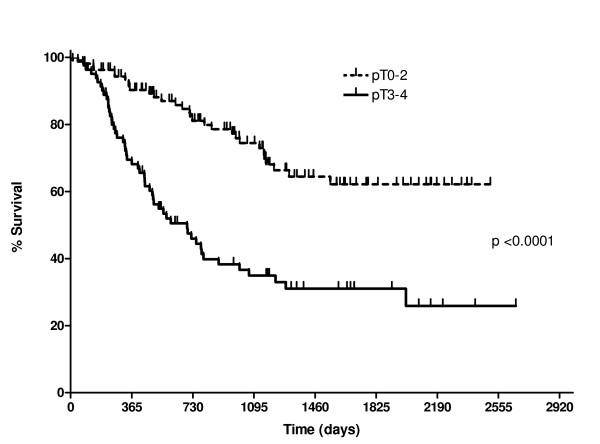
Survival curves comparing overall survival for p (and yp) T0–2 tumours versus p (and yp) T3–4 tumours.

**Figure 2:**
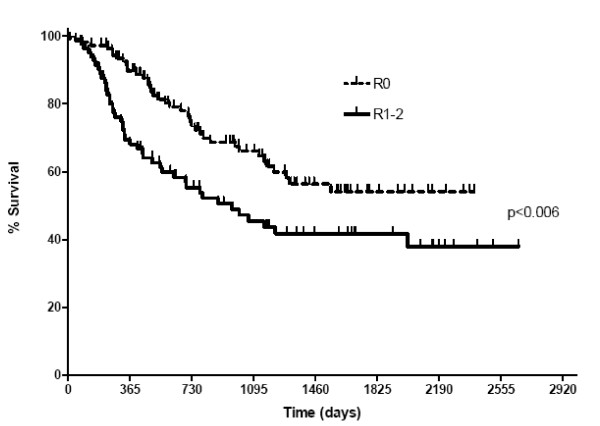
**Survival curves comparing overall survival for R0 and R1–2 resections. There was only one R2 resection**.

The radicality of resection in relation to tumour infiltration and involved lymph nodes is shown in Table 3. The median lymph node yield in all patients was 12 (range 1–52). Both tumour stage and radicality of resection were independent predictors of overall survival on univariate analysis (Figures 1 &2).

**Table 3:** Pathology results from 194 patients undergoing transhiatal esophagectomy for invasive malignancy.

	**N0**	**N+**	**R0**	**R1**	**R2**	**% R0 resections**
**T0**	3	2	5			100%
**T1**	35	7	41	1		98%
**T2**	23	38	41	20		68%
**T3**	25	52	19	57	1	25%
**T4**	1	5	1	6		17%

### Recurrence and survival

**Figure 3:**
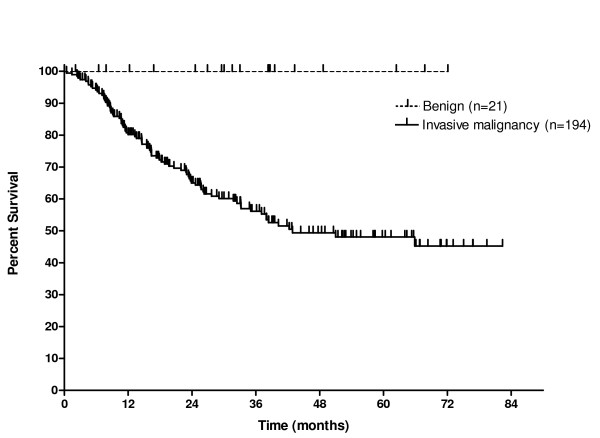
Kaplan Meier survival curves for overall survival of 21 patients with benign disease and 194 patients with invasive malignancy undergoing transhiatal esophagectomy.

All patients undergoing transhiatal esophagectomy for benign disease remain alive on follow up. Excluding the two in-hospital deaths, 79 patients (40%) who underwent esophagectomy for invasive malignancy have died on follow up. The causes of death are as follows: locoregional recurrence (n = 14), systemic metastases (n = 27), combination of locoregional recurrence and systemic metastases (n = 29), medical causes (n = 5), ongoing surgical complications (n = 1) and cause unable to be identified (n = 3). In total, 39% of patients developed recurrent disease during the period of study. The median survival for all patients undergoing transhiatal esophagectomy for invasive malignancy was 43 months and the one year and five year survival rates were estimated at 81% and 48% respectively (Figure 3). There was no difference in overall or disease free survival between patients with type I and II adenocarcinoma of the oesophagogastric junction.

## Discussion

This study has demonstrated that transhiatal esophagectomy can be associated with a low morbidity and a mortality of less than 1%. Although other units have reported similar results for transhiatal esophagectomy, several multicentre studies and national audits have shown that the mortality for all types of esophagectomy may exceed 10% [12-16]. It is recognised that high volume centres with a concentration of surgical, critical care and interventional radiological expertise achieve better outcomes. [17-19] The rationale for a transhiatal esophagectomy is the avoidance of a thoracotomy, thereby reducing the incidence of pulmonary complications, and the fashioning of a cervical anastomosis so that the clinical consequences of any anastomotic leak are minimized [12,13]. Critics of the transhiatal approach argue that there is a risk of blind intrathoracic injuries such as massive bleeding from the azygous vein, tracheal injury and episodes of cardiac instability resulting from retraction and surgical manipulation within the mediastinum. Case selection for transhiatal esophagectomy is crucial to prevent these problems and also to ensure adequate macroscopic tumor clearance for more proximally located esophageal tumors. It is the authors' policy that only patients with subcarinal tumors identified on preoperative imaging and confirmed by transhiatal dissection to above the proximal macroscopic extent of the tumor are suitable for the transhiatal approach. In the current series, only one patient required intraoperative conversion to a thoracotomy to obtain tumor clearance and 2 patients (1%) required reoperation for bleeding (both of these patients had active intrathoracic bleeding although none were associated with an azygous vein injury). Clinically apparent anastomotic leaks occurred in 6% of patients and all were managed successfully with conservative treatment. The data from this study supports the concept that a transhiatal esophagectomy in appropriately selected patients is safe and feasible.

Surgeons who advocate a transthoracic approach argue that neglecting to perform a mediastinal lymphadenectomy risks leaving behind residual tumour, resulting in higher rates of locoregional recurrence and worse overall survival. [20-22] However, the additional value of formal mediastinal lymph node dissection remains controversial in Western patients, especially with the concept that lymph node involvement may reflect systemic micrometastatic disease and that extended resections will not alter the natural history of this disease. Reported differences in recurrence and survival may merely represent a stage migration effect due to an increased accuracy of histological staging. [2,23,24] Portale et al recently suggested that extended en bloc transthoracic resections were significantly associated with better survival rates of up to 50% compared to transhiatal resections and that this could not be ascribed to a stage migration effect. [21] R0 status (defined in this study as clear circumferential and longitudinal margins) is a recognized independent prognostic factor for survival. Advocates of a transthoracic esophagectomy have suggested that the transhiatal approach limits the ability to achieve an R0 resection [20-22]. Macroscopic tumour clearance was achieved in all but one patient in the current study. Longitudinal margin involvement, especially at the proximal margin, has been shown to independently impact on survival via increased loco-regional recurrence. The rate of positive longitudinal margins in this study was 5% which is in keeping with other published series [25]. The problem of a positive gastric resection margin at transhiatal esophagectomy has recently been addressed by DiMusto and Orringer [26]. They achieved a negative gastric margin in 98% of over 1000 patients treated. In the few patients who had a positive gastric margin, they found that 80% die with distant metastases, which would not be influenced by more extensive gastric resection, and, in about 20%, local tumor recurrence in the intrathoracic stomach was usually asymptomatic. They also demonstrated that adjuvant therapy for a positive gastric margin was usually unhelpful. A similar picture was seen in the current study with all five patients with involved distal resection margins developing systemic metastases.

The role of circumferential resection margin (CRM) involvement is more controversial. Khan et al concluded that a positive CRM did not influence outcome. [27], but this has been disputed by other studies which suggested that it may independently predict survival [28]. One of these was performed by Maynard and colleagues who recently studied 242 patients undergoing esophagectomy and reported higher rates of local recurrence in patients with a positive CRM. Interestingly, there was no difference in CRM positivity when comparing different operative approaches [29].

In our population, CRM involvement was encountered in 46% of patients with malignant disease, predominantly affecting those with T3 tumours, and this was the main limiting factor in achieving an R0 resection. R0 resection rates varied from 97–100% with T0/1 tumours to 0–17% for T3–4 tumours. In keeping with previous studies, R0 resections were significantly associated with improved overall survival and hence the group benefiting most from this operative approach would appear to be those patients with early (T1–2) tumours. [20-22]

Advocates of more radical en-bloc transthoracic strategies argue that their approach may reduce rates of CRM involvement although this is yet to be proven [28]. Regardless of the operative technique, it is often difficult to obtain circumferential clearance due to the proximity of vital structures and the lack of any fascial boundaries. [13,28] The local recurrence rates in this study compare favourably to previous studies of both transhiatal and transthoracic esophagectomy [20,21,30,31]. Furthermore, the predominant pattern of recurrence was haematogenous metastatic disease (present in 70% of patients with disease relapse), mirroring the patterns seen with more radical en-bloc strategies [32]. These patterns of early systemic relapse were also noted by Orringer in his analysis of 2000 esophagectomy patients [33].

To date, there has been only one randomised controlled trial comparing transthoracic and transhiatal approaches and this failed to show any significant differences in radicality of surgery or survival at the cost of increased postoperative morbidity in the transthoracic group. [34] Recent five year survival data from this trial have again failed to demonstrate a survival benefit for the transthoracic approach although a sub-group of patients with oesophageal cancer and 1–8 involved lymph nodes appear to have improved disease-free survival. This study did not include chemotherapy and overall five year survival rates were 34% (Transhiatal) and 36% (Transthoracic) with in-hopsital mortality of 2% and 7% respectively [35]. Other meta-analyses have attempted to compare the two approaches and have favoured the transhiatal approach in terms of early morbidity and mortality with no long term survival disadvantage [22,36]. Despite this evidence, it remains difficult preoperatively to select the appropriate operative approach for individual patients.

Over the last few decades, the survival rates following esophagectomy have significantly improved, largely as a result of improvements in postoperative mortality. The one year survival rate of 81% in the current study for patients with invasive malignancy compares very favorably with the Western standard from the 1990s of 61%. [37] Furthermore, quality of life data suggests patients undergoing a transhiatal approach have fewer physical symptoms and better activity levels in the short term compared to the transthoracic approach although these differences become less evident by 1 year. [38] Several authors have emphasized the central role of surgery in achieving five year survival rates of approximately 50%. [21,30] It is increasingly recognized that there is an important role for oncological treatments in the perioperative management of esophageal and esophagogastric junctional cancer. The survival advantages associated with chemotherapy in both the MRC OEO2 and MRC MAGIC trials have significantly influenced surgical decision making in the UK. [3,39,40] The current series, which combined transhiatal esophagectomy with neoadjuvant chemotherapy in 42% of patients, has achieved equivalent five year survival results to Portale et al but with a greater preponderance of AJCC stage II and III disease. A complete pathological response was seen in 4% of patients receiving neoadjuvant chemotherapy and for many patients, there was little or no histological evidence of response. This emphasizes the need to identify potential responders prior to treatment, and also for the development of new chemotherapeutic agents. [21]

The development of high volume centres within the UK and the increasing use of (neo)adjuvant therapies have undoubtedly improved both the short term surgical results as well as the long term oncological outcomes of these patients. In summary, we have shown that transhiatal esophagectomy is a safe approach in appropriately selected patients. Radical resections, postoperative complication rates and survival results were in line with data reported for traditional transthoracic approaches. Some units restrict transhiatal esophagectomy to patients deemed unfit for thoracotomy or to patients with very early tumours or, conversely, locally advanced tumours where the benefits of more radical resections may be limited. However, the authors suggest that transhiatal esophagectomy is at least a viable alternative with certain advantages in terms of post-operative recovery, and ever improving oncological outcomes especially when combined with chemotherapy.

## Authors' contributions

AD was primary author of the manuscript. MF performed some of the surgery, set up the database and assisted in data collection as well as drafting of the paper. AK, VP and AN were the primary data collectors and also performed the statistical analysis. DS helped conceive the study, performed some of the surgery and assisted in data collection. RM was the consultant in charge, performed the majority of the surgery and made alterations to the final draft prior to submission. All authors read and approved the final manuscript.
